# Infliximab therapy increases body fat mass in early rheumatoid arthritis independently of changes in disease activity and levels of leptin and adiponectin: a randomised study over 21 months

**DOI:** 10.1186/ar3169

**Published:** 2010-10-21

**Authors:** Inga-Lill Engvall, Birgitta Tengstrand, Kerstin Brismar, Ingiäld Hafström

**Affiliations:** 1 Department of Rheumatology, Karolinska Institutet at Karolinska University Hospital Huddinge, R92, Stockholm 141 86, Sweden; 2 Department of Molecular Medicine and Surgery, Rolf Luft Research Center for Diabetes and Endocrinology at Karolinska Institutet, Karolinska University Hospital, Solna L1:00, Stockholm 171 76, Sweden

## Abstract

**Introduction:**

Rheumatoid arthritis (RA) is associated with changes in body composition and bone mineral density (BMD). The purpose of the present study was to evaluate whether anti-TNF treatment in early RA has an impact on body composition and BMD besides that which could be achieved by intensive disease-modifying anti-rheumatic drug (DMARD) combination therapy.

**Methods:**

Forty patients with early RA who failed treatment with methotrexate up to 20 mg/week for 3 months were randomised to addition of sulphasalazine and hydroxychloroquine (treatment A) or addition of infliximab (treatment B). At 3, 12 and 24 months, body composition and BMD were assessed by total-body dual-energy X-ray absorptiometry. At the same time points, leptin, adiponectin, apolipoproteins, insulin-like growth factor-1 (IGF-1) and markers of bone remodelling were analysed. Compliance to treatment was considered in the analyses. Data were analysed with a mixed, linear model.

**Results:**

Patients treated with anti-TNF had a significant increase in fat mass at 2 years, 3.8 (1.6 to 5.9) kg, in contrast to patients in treatment A, 0.4 (-1.5 to 2.2) kg (*P *= 0.040), despite similar reduction in disease activity. Both treatment strategies prevented loss of muscle mass and bone. Leptin concentrations increased significantly in both groups at 2 years and adiponectin increased significantly at 2 years in treatment A and at 1 year in treatment B. There were no significant changes in apolipoproteins or IGF-1. The markers of bone resorption decreased at 12 months in both treatment groups with no significant difference between the treatment groups.

**Conclusions:**

Infliximab therapy increased body fat mass, an effect that was not achieved with the combination of DMARDs, despite a similar reduction in disease activity, and thus seemed to be drug specific. The increase of fat mass was not associated with an exacerbated atherogenic lipid profile. Leptin and adiponectin concentrations increased in both treatment groups. The increase of adiponectin may partially explain the reduced frequency of cardiovascular diseases found when disease activity is reduced in RA.

**Trial registration:**

ISRCTN39045408.

## Introduction

Rheumatoid arthritis (RA) is a chronic inflammatory disease associated with changes in body composition [1] and decreased bone mineral density (BMD) [2].

The change in body composition with loss of skeletal muscle mass and accumulation of fat is known as rheumatoid cachexia and is associated with increased disability, morbidity and mortality [1,3]. Increased fat mass, especially abdominal fat mass, thus increases the risk for type 2 diabetes and cardiovascular diseases (CVD) [4]. CVD has turned out to be one of the most important causes of death in RA patients [5]. Furthermore, loss of body protein is associated with muscle weakness and impaired adaptation to metabolic stress also affecting morbidity and mortality [1]. The combination of fat mass gain and reduced muscle mass may compound these individual risks [6].

The changes in body composition and BMD have been regarded to be consequences of the catabolic process induced by the chronic inflammatory disease and especially attributed to proinflammatory cytokines, like TNFα, and physical inactivity [1]. If so, treatments that reduce inflammation should normalise the deranged body composition and hamper bone loss. In this context it is especially interesting to investigate whether treatment with TNF antagonists, which powerfully reduces disease activity in RA [7], might have this potency.

Recently, treatment with anti-TNF in patients with early as well as longstanding RA has been reported not to affect body composition [8-10]. The treatment periods were only 3 to 12 months, however, which probably is too short a time to detect significant changes. This suggestion is strengthened by the fact that anti-TNF treatment during 2 years in patients with spondyloarthropathy resulted in significant increase in body weight mainly due to gain in fat mass [11]. A possibility is that the new therapeutic strategies contribute to body fat gain by controlling weight loss in patients that still have decreased physical activity [12]. The study on spondyloarthropathy was uncontrolled and could not differentiate between a specific effect of TNF antagonists and a general effect of reduced inflammatory activity [11].

The primary objective of the present study was to investigate whether infliximab had any effects on body composition and BMD beyond the anti-inflammatory effect in patients with early RA. The patients were randomised to intensive treatment with methotrexate (MTX) in combination with sulphasalazine and hydroxychloroquine or to MTX in combination with infliximab. The patients were analysed regarding changes in body composition and BMD after 9 and 21 months, considering whether they were compliant to their respective treatment or not. Secondary objectives were to analyse whether infliximab affected levels of adipokines and apolipoproteins, substances of importance for CVD, as well as levels of biomarkers of bone metabolism and insulin-like growth factor-1 (IGF-1), and whether such changes were related to changes in body composition and BMD.

## Materials and methods

### Patients

Forty out of 44 consecutive patients with early RA who participated in the Swefot (Swedish Pharmacotherapy) study at Karolinska University Hospital were included between April 2004 and October 2005.

Swefot is an open, multicentre, randomised study designed to compare two treatment strategies in patients in whom MTX up to 20 mg/week had not lowered the Disease Activity Score of 28 joints (DAS28) to ≤3.2 during the first 3 months of disease treatment [13]. After 3 months, the patients who had not achieved low disease activity were randomised by block randomisation for each centre according to a central randomisation programme: treatment A, MTX with addition of sulphasalazine 2,000 mg/day and hydroxychloroquine 400 mg daily; and treatment B, MTX with the addition of the TNF antagonist infliximab 3 mg/kg body weight given intravenously at weeks 0, 2 and 6 and every 8 weeks thereafter. The patients had RA according to the American College of Rheumatology criteria [14], had disease duration <12 months and had active disease defined as DAS28 >3.2.

**Figure 1:**
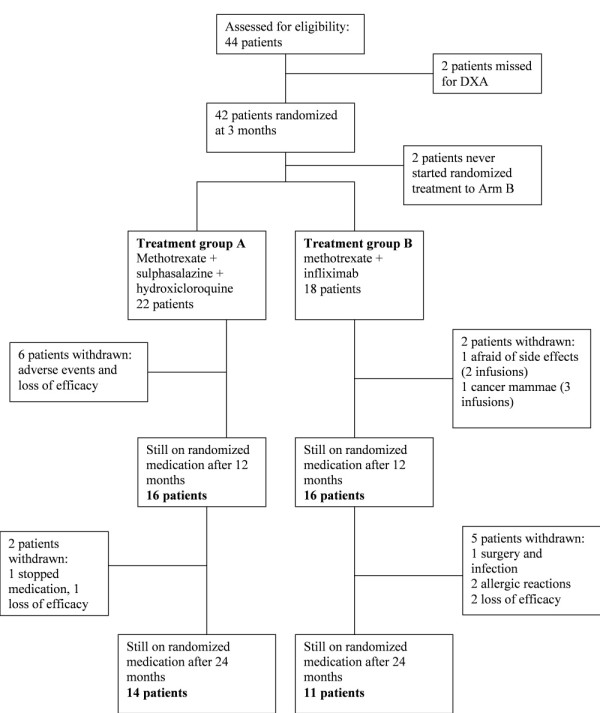
**Distribution of the study patients by treatment group**. DXA, Dual X-ray absorptiometry.

Forty patients were eligible to inclusion in this substudy as they were assessed by dual X-ray absorptiometry measurements and had started treatment according to the randomisation (Figure 1). Of these patients, 22 had been randomised to treatment A and 18 to treatment B. At 24 months, two of the patients with treatment B had changed from infliximab to etanercept. The reasons for withdrawal from the protocol treatment are shown in Figure 1. Three of the evaluated patients (one patient with treatment A and two patients with treatment B) received treatment with glucocorticoids (5 to 7.5 mg daily), and two patients were treated with bisphosphonates, both with treatment B. Three patients were treated with lipid-lowering drugs, one with treatment A and two with treatment B.

The study was approved by the Karolinska University Hospital ethics committee and was performed in accordance with the Helsinki Declaration. All patients received verbal and written information on the trial and signed a consent form prior to inclusion. The trial is registered in the ISRCTN register (number ISRCTN39045408).

### Clinical assessments

Disease activity was assessed by the DAS28 [15], and functional status was measured using the Swedish version of the Stanford Health Assessment Questionnaire (HAQ) [16].

### Body composition assessments

The body mass index (BMI) was calculated and defined as low (<18.5 kg/m^2^), normal (18.5 to 24.9 kg/m^2^) or high (>25 kg/m^2^) [17]. Body composition was measured by dual X-ray absorptiometry with a densitometer (GE-Lunar Progedy, Maddison, MA, USA). Fat mass (FM), lean body mass (LBM), bone mineral content and BMD were assessed at the 3-month, 1-year and 2-year visits. The appendicular lean mass was calculated by adding lean mass from both arms and legs. Fat-free mass (FFM), the sum of LBM and bone mineral content, was expressed in absolute kilograms, and the fat mass index (FMI) as well as the fat-free mass index (FFMI) were calculated [18]. Fat from arms and legs were summed to estimate peripheral fat, and a trunk/peripheral fat ratio was created by dividing trunk regional fat by peripheral regional fat.

BMD values were given in grams of bone mineral per square centimetre, as *T *scores (the number of standard deviations (SDs) from the mean in healthy young sex-matched people) and as *Z *scores (the number of SDs from the mean of healthy age-matched and sex-matched people); values obtained from the Lunars combined European/US reference population [19].

### Body composition definitions

The reference value for FM percentage is 20 to 30% for women and 12 to 20% for men [20]. Overweight is defined as FM > 33% for women and FM > 25% for men. Obesity for persons younger than 60 years is defined as FM > 41% for women and FM > 29% for men, and for persons older than 60 years as FM > 43% for women and FM > 31% for men [21].

Trunk/peripheral fat ratios for healthy Swedish women and men are mean (95% confidence interval) of 0.91 (0.87, 0.95) and 1.34 (1.25, 1.44), respectively [22]. A higher trunk/peripheral fat ratio indicates central obesity. Data from a Swiss population of healthy adults (2,986 men, 2,649 women) were used to classify the patients as underlean or having excess FM [18]. The cut-off point for low muscle mass was defined as a FFMI below the 10th percentile and obesity was defined as a FMI above the 90th percentile. Underlean implies muscle wasted (that is, insufficient muscle mass).

Osteoporosis was defined as a *T *score ≤-2.5 SDs [23] and osteopenia as a *T *score <-1.0 SD from the mean in healthy, young, sex-matched people.

### Assays on blood samples

Serum samples were obtained between 9:00 am and 3:00 pm and were stored at -70°C until all samples were analysed. Leptin, adiponectin and IGF-1 were analysed at the Rolf Luft Center for Diabetes Research, Department of Molecular Medicine and Surgery, Karolinska Institutet, and apolipoproteins and bone markers were analysed at the Study Centre for Laboratory Medicine, Karolinska University Hospital, both in Stockholm, Sweden.

Leptin was determined by radioimmunoassay using HL-81K (Linco Research, Inc, St Charles, Missouri, USA). Normal mean (SD) leptin values (BMI ranges 18 to 25) are for lean men 3.8 (1.8) μg/l and for lean women 7.4 (3.7) μg/l. Levels rise approx 2.5 times faster in women per unit BMI as compared with men [24].

Adiponectin was determined by radioimmunoassay using HADP-61 (Linco Research, Inc, St Charles, Missouri, USA). The sensitivity of the assay is 1 ng/ml. Mean (SD) levels from 205 Swedish healthy adults (106 women, 99 men) are for women 11.88 (4.69) mg/l and for men 7.34 (3.55) mg/l (AL Unden and K Brismar, personal communication). The intra-assay precision coefficient of variation (CV) is 3.86% and the inter-assay precision CV 8.47%.

Apolipoprotein A1 (apoA1) and apolipoprotein B (apoB) were determined by Synchrone LX from Beckman AB (Fullerton, California, USA) using turbidimetry. The reference intervals for apoA1 and apoB were given by the manufacturer. The apoA1 reference intervals are for women 1.10 to 2.10 g/l and for men 1.10 to 1.80 g/l. The apoB reference interval was determined by Synchrone LX from Beckman AB using turbidimetry. The reference interval for individuals younger than 40 years is 0.50 to 1.50 g/l and for individuals 40 years and older is 0.50 to 1.70 g/l.

The apoB/apoA1 ratio was calculated. A ratio >0.6 for women and >0.7 for men is considered a moderate risk for CVD, and the risk increases almost linearly with increasing ratio. For patients with other risk factors for CVD, the preferable ratio should be even lower [25].

The bone markers analysed were procollagen type I N-terminal propeptide (P1NP) as a marker of bone formation, and C-terminal telopeptide crosslaps (CTX-1) as well as C-terminal telopeptides of type I collagen (1CTP) as markers of bone degradation. Reference intervals for the bone markers were given by the manufacturer.

P1NP was determined by the Elecsys 1010/2010 total P1NP serum kit from Roche Diagnostics (Mannheim, Germany), which employs the electrochemiluminescence immunoassay technique. The measuring range is 5 to 1,200 μg/l. The reference intervals are for premenopausal women <60 μg/l, for postmenopausal women <80 μg/l and for men <45 μg/l. The intra-assay CV is 2.3% and the total precision CV is 2.9%.

CTX-1 was determined by the Elecsys 1010/2010 β-CrossLaps serum kit from Roche Diagnostics, which also employs the electrochemiluminescence immunoassay technique. The measuring range is 10 to 6,000 pg/ml. The sensitivity of the assay is 0.01 ng/ml. The reference intervals are for premenopausal women <570 pg/ml and for postmenopausal women <1,000 pg/ml, and for men younger than 50 years of age <580 pg/ml, for men between 50 and 70 years old <700 pg/ml and for men older than 70 years <850 pg/ml. The intra-individual CV is 17.9%. The intra-assay CV is 2.4% and the total precision CV 3.1%.

1CTP was determined by the Multigamma radioimmunoassay kit from Orion Diagnostica (Espoo, Finland). The sensitivity of the assay is 0.5 μg/l. The reference interval is 1.8 to 5.0 μg/l. The intra-assay CV and the inter-assay CV are 5.3% and 4.25%, respectively.

The anabolic factor IGF-1 was determined by radioimmunoassay, after separation of insulin-like growth factor from its binding proteins by acid ethanol extraction and cryoprecipitation. To minimise interference of remaining insulin-like growth factor binding proteins, des(1-3)-IGF-1 was used as the radioligand [26]. The intra-assay and inter-assay CVs were 4% and 11%. As serum levels of IGF-1 are age dependent, decreasing with age, IGF-1 values were also expressed as age-specific SD scores calculated from the regression of the values of 247 healthy adult subjects [27].

### Statistical analysis

All eligible patients, regardless of compliance with the protocol, were included in the analyses. STATISTICA release 8 (Stat Soft Scandinavia AB, Tulsa, OK, USA) and SAS System 9.1 (SAS Institute Inc., Cary, NC, USA) were used for statistical analyses. Data are presented as mean (SD) or median (interquartile range) depending on their distribution. Correlation analysis was performed with Spearman rank order correlations. Area under the curve (AUC) analyses were calculated according to the trapezoidal rule and included values from time points 0, 3, 12 and 24 months. Data were analysed using a mixed linear model [28] with two between-group factors - Treatment (A and B) and Compliance (<24 months and 24 months) - and one within-group factor - Time (3 months, 12 months and 24 months). Different covariance models were tested and the covariance structure with the smallest value of the Akaike's Information Criterion (AICC and BIC) was considered to best fit the data. In case of significant interactions, simple main effect tests were performed; that is, the effects of one factor holding the level of the other factors fixed. Results are presented as mean (standard error) and 95% confidence interval. The distribution of some variables was positively skewed and before the formal analyses the variables were log-transformed. *P *< 0.05 was considered statistically significant.

## Results

At baseline and the 3-month visit, the time for randomisation, the two treatment groups were well balanced as regards demographic and clinical variables (Table 1).

**Table 1:** Demographic, clinical and laboratory characteristics for patients at baseline and at the 3-month visit (time for randomisation)

	Treatment A (*n *= 22)	Treatment B (*n *= 18)
Baseline		
Age (years)	59.5 (58.0 to 67.0)	56.0 (42.0 to 73.0)
Women	16 (73%)	13 (72%)
Menopause	14 (88%)	8 (62%)
Current or previous smoker	15(68%)	7 (41%)
Disease duration (months)	5.4 (2.9)	4.9 (3.4)
Rheumatoid factor positivity	11 (50%)	10 (56%)
X-ray criteria at baseline	7 (32%)	2 (11%)
3-month visit		
Erythrocyte sedimentation rate (mm)	25.0 (14.0 to 29.0)	23.5 (12.0 to 34.0)
Disease Activity Score of 28 joints	4.3 (4.1 to 4.9)	4.8 (3.7 to 5.1)
HAQ score	0.86 (0.50)	0.89 (0.18)
Leptin (μg/l)	8.8 (5.4 to 27.7)	7.9 (3.9 to 14.2)
Adiponectin (mg/l)	10.7 (3.4)	10.9 (6.6)
Apolipoprotein A1 (g/l)	1.46 (0.25)	1.64 (0.23)
Apolipoprotein B (g/l)	1.10 (0.26)	1.03 (0.21)
Apolipoprotein B/apolipoprotein A1	0.77 (0.24)	0.64 (0.15)
P1NP (μg/l)	46 (36 to 58)	43 (32 to 68)
CTX-1 (pg/ml)	280 (191 to 368)	233 (119 to 457)
1CTP (μg/l)	4.1 (3.3 to 5.6)	3.7 (2.9 to 4.9)
IGF-1 (μg/l)	145.5 (114.0 to 166.0)	154.5 (130.0 to 193.0)
IGF-1 SD scores	-0.09 (1.34)	-0.07 (-0.12)

Demographic, clinical and laboratory characteristics at baseline and at the 3-month visit (time for randomisation). Data presented as median (interquartile range), *n *(%) or mean (standard deviation (SD)). Treatment A, methotrexate + sulphasalazine + hydroxychloroquine; treatment B, methotrexate + infliximab. HAQ, Health Assessment Questionnaire, P1NP, procollagen type I N-terminal propeptide; CTX-1, C-terminal telopeptide of type I collagen; 1CTP, C-terminal propeptide of type I collagen; IGF-1, insulin-like growth factor-1.

### Body mass index and body composition

BMI and body composition data at the 3-month visit are presented in Table 2. At the 3-month visit, 50% of the patients had a BMI corresponding to overweight (BMI > 25 kg/m^2^). Of the women, 76% had a FM percentage corresponding to overweight and 24% corresponding to obesity. The figures for men were 91% and 64%, respectively. Twenty percent of the patients (seven women, one man) had low muscle mass, FFMI below the 10th percentile of the reference population.

**Table 2:** Body composition and bone mineral density at the 3-month visit (time for randomisation)

	Treatment A (*n *= 22)	Treatment B (*n *= 18)
Body mass index (kg/m^2^)	27.7 (6.4)	24.7 (3.7)
Fat mass (kg)	30.5 (12.9)	24.4 (7.5)
Fat mass index (kg/m^2^)	10.9 (4.8)	8.7 (2.9)
Fat (%)	38.8 (8.3)	35.7 (7.9)
Trunk:peripheral fat ratio	1.3 (0.3)	1.2 (0.4)
Trunk fat (kg)	16.4 (7.2)	13.9 (6.6)
Lean body mass (kg)	45.8 (8.8)	43.4 (9.0)
Appendicular lean mass (kg)	19.9 (4.4)	18.7 (4.4)
Fat-free mass (kg)	48.3 (9.1)	45.9 (9.4)
Fat-free mass index (kg/m^2^)	17.0 (2.5)	16.1 (2.1)
L2 to L4		
Bone mineral density (g/cm^2^)	1.18 (0.19)	1.06 (0.14)
*Z *score	0.68 (1.40)	-0.66 (0.89)
*T *score	-0.31 (1.55)	-1.26 (1.18)
Femoral neck		
Bone mineral density (g/cm^2^)	0.98 (0.16)	0.92 (0.11)
*Z *score	0.50 (1.04)	-0.16 (0.79)
*T *score	-0.32 (1.25)	-0.82 (0.95)

Data presented as mean (standard deviation). Treatment A, methotrexate + sulphasalazine + hydroxychloroquine; treatment B, methotrexate +infliximab.

Changes in body composition and BMD are presented in Table 3. The patients compliant to treatment A had at 12 months a significant increase in FFM compared with the 3-month visit, an increase that was still present at 24 months.

**Table 3:** Changes in body composition and bone mineral density between 3 and 24 months

			*P *value	
			
	Treatment A (*n *= 14)	Treatment B (*n *= 11)	Time	Treatment × Compliance × Time
Body mass index (kg/m^2^)	0.54 (-0.37 to 1.44)	0.89 (-0.13 to 1.91)	0.07	0.37
Fat mass (kg)	0.36 (-1.54 to 2.25)	**3.77 (1.63 to 5.90)**	**0.028**	**0.040**
Fat mass index (kg/m^2^)	0.05 (-0.62 to 0.72)	**1.24 (0.48 to 2.00)**	**0.035**	**0.035**
Fat (%)	0.15 (-2.03 to 1.73)	**2.80 (0.67 to 4.91)**	0.11	0.17
Trunk/peripheral fat ratio	0.15 (-0.05 to 0.36)	0.18 (-0.05 to 0.42)	0.18	0.82
Trunk fat (kg)	-0.01 (-1.26 to 1.25)	1.11 (-1.95 to 4.17)	0.40	0.36
Lean body mass (kg)	1.12 (-0.06 to 2.30)	0.52 (-0.81 to 1.86)	**0.007**	0.79
Appendicular lean mass (kg)	0.08 (-0.65 to 0.80)	0.25 (-0.56 to 1.07)	0.26	0.83
Fat-free mass	**1.23 (0.08 to 2.38)**	0.57 (-0.73 to 1.87)	**0.004**	0.69
Fat-free mass index	0.29 (-0.15 to 0.72)	0.16 (-0.33 to 0.65)	**0.011**	0.63
*L2 *to *L4*				
BMD (g/cm^2^)	0.02 (-0.01 to 0.05)	0.03(0.00 to 0.06)	0.15	0.56
*Z *score	**0.31 (0.06 to 0.57)**	0.29 (0.00 to 0.58)	**0.030**	0.26
*T *score	0.26 (-0.03 to 0.55)	0.26 (-0.03 to 0.55)	0.20	0.55
BMD (g/cm^2^)	0.00 (-0.02 to 0.02)	0.01 (-0.03 to 0.01)	0.19	0.63
*Z *score	0.12 (-0.06 to 0.30)	0.04 (-0.24 to 0.17)	0.24	0.63
*T *score	0.01 (-0.14 to 0.17)	0.12 (-0.29 to 0.06)	0.20	0.32

Changes in body composition and bone mineral density (BMD) between 3 and 24 months, using a mixed linear model. Data presented as mean (95% confidence interval). Treatment A, methotrexate + sulphasalazine + hydroxychloroquine; treatment B, methotrexate +infliximab. Changes between 3 and 12 months and patients not compliant to respective treatment are not presented. *P *values given for the interactions with the main factor Time and the interactions with factor Time. Bold values are statistically significant.

The patients compliant to treatment B had a significant increase in FM and FMI at both 12 and 24 months, compared with the 3-month visit.

When comparing the treatment groups, there were no statistical significant differences in changes of BMI or body composition from 3 to 12 months. The changes from 3 to 24 months, however, were significantly different between the treatment groups as regards the increase in FM and FMI (*P *= 0.040 and *P = *0.035, respectively), where FM increased mean (SD) 3.4 (1.4) kg more in the patients in group B compared with patients in group A. Changes in LBM and FFM did not differ significantly between the treatment groups.

### Bone mineral density

At the 3-month visit, 10% of the patients had osteoporosis and 48% osteopenia.

The patients compliant to treatment A had at 24 months a significant increase in *Z *score at lumbar spine, compared with the 3-month visit (Table 3). The patients compliant to treatment B had a trend for an increase in BMD and *Z *score at the lumbar spine after 24 months. There were no significant changes at the femoral neck between 3 and 24 months in either treatment group. There were no significant differences between the treatment groups in changes in bone mineral at either skeletal site between the randomisation and the 24-month follow-up (Table 3).

### Inflammatory activity and disability

Table 4 presents the results after 24 months of randomised treatment. The DAS28 and the HAQ score decreased during the study period in both treatment groups and there were no significant differences in changes in inflammatory activity or disability between the treatment groups.

**Table 4:** Changes in different variables between 3 and 24 months, using a mixed linear model

			*P *value	
			
	Treatment A (*n *= 14)	Treatment B (*n *= 11)	Time	Treatment × Compliance × Time
DAS28	**-1.8 (-2.6 to -1.0)**	**-1.9 (-2.8 to -1.0)**	**<0.001**	0.59
ESR^**a**^	**-0.62 (-1.07 to -0.18)**	-0.49 (-0.99 to 0.02)	**0.003**	0.78
HAQ	**-0.27 (-0.50 to -0.04)**	**-0.43 (-0.69 to -0.17)**	**<0.001**	0.82
Leptin^**a**^	**0.25 (0.02 to 0.48)**	**0.32 (0.05 to 0.59)**	**<0.001**	0.07
Adiponectin	**1.66 (0.16 to 3.17)**	2.33 (-0.27 to 4.93)	**<0.001**	0.058
Apolipoprotein A1	0.09 (-0.08 to 0.26)	0.19 (-0.01 to 0.39)	**0.004**	0.45
Apolipoprotein B	-0.06 (-0.16 to 0.03)	0.07 (-0.04 to 0.18)	0.59	0.33
ApoB/ApoA1	0.09 (-0.19 to 0.00)	-0.01 (-0.12 to 0.10)	**0.023**	0.56
P1NP^**a**^	-0.02 (-0.030 to 0.25)	-0.29 (-0.61 to 0.03)	**0.004**	0.82
CTX1^**a**^	-0.25 (-0.64 to 0.14)	-0.47 (-0.62 to 0.28)	**<0.001**	0.11
1CTP^**a**^	-0.05 (-0.27 to 0.16)	-0.21 (-0.46 to 0.04)	**<0.001**	0.76
P1NP/CTX-1^**a**^	0.23 (-0.04 to 0.49)	-0.10 (-0.41 to 0.21)	**0.023**	0.08
P1NP/1CTP^**a**^	0.03 (-0.28 to 0.34)	-0.08 (-0.44 to 0.28)	0.45	0.56
IGF-1^**a**^	0.06 (-0.07 to 0.18)	-0.02 (-0.17 to 0.13)	**0.050**	0.08
IGF-1 SD score	0.32 (-0.14 to 0.78)	0.02 (-0.52 to 0.56)	**0.003**	0.12

Data presented as mean (95% confidence interval). ^a^Arithmetic mean. Treatment A, methotrexate + sulphasalazine + hydroxychloroquine; treatment B, methotrexate +infliximab. DAS28, Disease Activity Score of 28 joints; ESR, erythrocyte sedimentation rate; HAQ, Health Assessment Questionnaire; apo, apolipoprotein; P1NP, procollagen type I N-terminal propeptide; CTX-1, C-terminal telopeptide of type I collagen; 1CTP, C-terminal propeptide of type I collagen; IGF-1, insulin-like growth factor-1; SD, standard deviation. Changes between 3 and 12 months and patients not compliant to respective treatment are not presented. *P *values given for the interactions with the main factor Time and the interactions with factor Time. Bold values are statistically significant.

After 12 months of treatment, 38% of patients compliant to treatment A and 25% of patients compliant to treatment B had received remission (DAS28 < 2.6). The frequencies after 24 months were 50% and 73%, respectively. There was no significant difference between the treatment groups in remission frequency at either time point.

### Leptin

At randomisation, the leptin levels for lean women were median (interquartile range) 5.9 (3.1 to 13.3) μg/l whereas for lean men the levels were 3.1 (2.7 to 4.7) μg/l. When including all patients, irrespective of BMI, the levels were median (interquartile range) 13.9 (5.5 to 23.9) μg/l for women and 5.4 (3.1 to 6.8) μg/l for men.

Leptin increased significantly from 3 to 24 months in treatment group A, whereas in treatment group B there was a significant increase from 3 to 12 months as well as from 3 to 24 months. There was no significant difference in changes in leptin levels between the treatment groups (Table 4).

### Adiponectin

At randomisation, the adiponectin level for women was mean (SD) 11.49 (5.29) mg/l. For men, the level was 8.84 (3.65) mg/l.

Adiponectin increased significantly from 3 to 12 months as well as from 3 to 24 months in patients compliant to treatment A, whereas in patients compliant to treatment B there was a significant increase only between 3 and 12 months There was no significant difference in changes in adiponectin levels between the treatment groups (Table 4).

### Apolipoproteins

At randomisation, the apolipoproteins were in the reference intervals: apoA1 mean (SD) 1.58 (0.26) for women and 1.42 (0.23) for men, and apoB 0.90 (0.20) for patients younger than 40 years of age and 1.08 (0.23) for those older than 40 years.

There was a trend for an increase in apoA1 between 3 and 24 months in patients compliant to treatment B (Table 4). Change in apoA1 was positively correlated with the increase in adiponectin from 3 to 24 months (*r *= 0.38, *P *= 0.017). There was no significant change in apoB during the study period.

At 3 months the apoB/apoA1 ratio was mean (SD) 0.68 (0.22) for women and 0.80 (0.19) for men. There was a trend for a decrease in the apoB/apoA1 ratio in patients compliant to treatment A, while the ratio remained unchanged in patients compliant to treatment B (Table 4). There were no significant differences in changes of the apolipoproteins or in apoB/apoA1 ratio between the treatment groups.

### Markers of bone turnover

After 12 months there was a significant decrease in 1CTP in patients compliant to treatment A (*P *= 0.034) and a trend for a decrease in CTX-1 (*P *= 0.060). For patients compliant to treatment B there was a significant decrease in CTX-1 (*P *= 0.017) and a trend for a decrease in 1CTP (*P *= 0.060). There was no significant change in P1NP between 3 and 12 months but there was a trend for a decrease between 3 and 24 months in treatment group B (*P *= 0.08). The P1NP/CTX-1 ratio increased significantly between 3 and 12 months in treatment group B (*P *= 0.032) and there was a trend for an increase also in treatment group A (*P *= 0.09).

The changes in markers of bone resorption and formation between 3 and 24 months are shown in Table 4. There were no significant changes in markers of bone resorption between 3 and 24 months, and no significant difference in changes in markers of bone turnover or ratios of bone markers between the treatment groups during the study period (Table 4).

### Anabolic factor IGF-1

The mean IGF-1 at baseline corresponded to a mean IGF-1 SD score within the normal range of the reference population. IGF-1 remained unchanged during the study period and there was no difference in change in IGF-1 between the treatment groups (Table 4).

### Correlations between body composition and BMD and clinical and laboratory variables at randomisation for all patients

Leptin correlated positively and significantly with most variables of fat (FM *r *= 0.78, *P *< 0.001). Adiponectin was significantly negatively correlated with the trunk/peripheral fat ratio (*r *= -0.42, *P *= 0.006). There were no significant correlations between FM and disease duration, markers of inflammation, HAQ score, apolipoproteins or IGF-1. There was a positive correlation between age and FMI (*r *= 0.33, *P *= 0.040).

There were no significant correlations between LBM, appendicular lean mass, FFM or FFMI and age, disease duration, markers of inflammation, HAQ score or IGF-1.

There was a positive correlation between leptin and *Z *score at the lumbar spine (*r *= 0.39, *P *= 0.015). There were no other significant correlations between BMD at the lumbar spine or total hip and disease duration, markers of inflammation, HAQ score, IGF-1 or markers of bone turnover.

### Correlations between changes in body composition and BMD and AUC of different variables

The AUC values were calculated from the values at time points 0, 3, 12 and 24 months.

High AUC values for apoB and the apoB/apoA1 ratio were associated with increased trunk/peripheral fat ratio (*r *= 0.43, *P *= 0.006 and *r *= 0.46, *P *= 0.003, respectively). All fat variables, except the trunk/peripheral fat ratio, were significantly positively correlated with adiponectin - the highest correlation coefficient being with fat percentage (*r *= 0.39, *P *= 0.013). AUC values for the erythrocyte sedimentation rate, DAS28, HAQ score, IGF-1, leptin and apolipoproteins did not significantly correlate with changes in FM, FMI, trunk fat or peripheral fat.

AUC values for the erythrocyte sedimentation rate, DAS28, HAQ score and IGF-1 did not significantly correlate with changes in LBM, appendicular lean mass, FFM or FFMI.

There were significant correlations between the AUC for P1NP and changes in BMD at the lumbar spine and femoral neck (*r *= -0.34, *P *= 0.040 and *r *= -0.35, *P *= 0.031, respectively), whereas there were no significant correlations between markers of bone resorption and BMD. AUC values for the erythrocyte sedimentation rate, DAS28, HAQ score, leptin and IGF-1 did not significantly correlate with changes in BMD at lumbar spine or femoral neck.

## Discussion

In this prospective randomised study of early RA followed during 2 years, patients treated with infliximab had a significant increase in FM. That was not the case in patients treated with MTX in combination with sulphasalazine and hydroxychloroquine, despite a similar reduction of disease activity. Both treatment strategies prevented loss of skeletal muscle mass and bone.

The analyses showed a specific effect of anti-TNF treatment on body composition with an increase of FM, an effect that has not been shown previously in RA with shorter treatment periods [8-10]. In spondylarthropathy, however, patients gained fat during 2 years of treatment with TNF antagonists [11] - but being without a control group, clarifying the role of anti-TNF versus control of the inflammatory activity was impossible. The lack of fat gain in the present patients on the combination treatment A therapy, despite a similar reduction of disease activity, indicates that the fat gain in the anti-TNF-treated patients was drug specific.

Adipose tissue secretes a variety of biologically active proteins, called adipokines, including leptin, adiponectin and TNFα [29]. Plasma leptin levels are reported to directly correlate with degree of adiposity in healthy humans [30]. Such a correlation was also present in the RA patients at the 3-month visit. The leptin AUC for 0 to 24 months did not correlate with changes in FM during 3 to 24 months, however, probably dependent on the fact that leptin levels increased in both treatment groups but FM increased only in the anti-TNF-treated patients. This hypothesis suggests a disruption between leptin levels and adiposity secondary to inflammation and/or anti-TNF therapy. In chronic inflammation, such as RA, leptin levels have been shown to be negatively correlated with inflammation [31]. This finding could explain the increasing levels seen when disease activity decreased and may also explain why leptin increased in treatment group A despite stable weight.

Anti-TNF therapy over 3 and 6 months has earlier been reported not to alter leptin concentrations [32,33], which is in contrast to the increase found here. This difference could possibly be ascribed to different lengths of therapy. Three months and 6 months may be too short a time to obtain a significant increase in FM, which is the main determinant of circulating levels of leptin.

Adiponectin increased in both treatment groups and was positively correlated with FM gain during the 21 months of treatment. This increase of adiponectin is opposite to the decreased levels found in non-RA individuals with obesity, which is a consequence of downregulation of adiponectin in obesity. Adiponectin has among other effects direct insulin-sensitising activity and a protective role in the development of CVD [34,35]. Increase of adiponectin found here when disease activity decreased might be one explanation for the reduced frequency of CVD reported in MTX-treated RA patients [36] as well as during anti-TNF therapy [37]. The evidence that adiponectin has atheroprotective effects in RA is further supported by the improved endothelial function in parallel with increasing adiponectin levels during anti-TNF therapy [38].

The effect of TNF antagonists on adiponectin levels is contradictory. Both stable levels [32,39,40] and increasing levels [10,38,41] have thus been reported in RA patients receiving anti-TNF. The explanation for this discrepancy is unclear, but might depend on different patient populations as well as varying anti-TNF preparations. Treatment with MTX has also been shown to increase the level of adiponectin [42]. Patients with different inflammatory diseases have shown elevated levels of adiponectin, and it is speculated that the elevated adiponectin is a counteracting mechanism to protect from harmful effects of different diseases [43].

To investigate whether the gain in FM was associated with an atherogenic lipid profile, we also analysed apoA1 and apoB. The apoB/apoA ratio at baseline indicated an increased risk of CVD for both genders. The ratio remained unchanged in treatment group B, whereas in treatment group A there was a trend for a decrease, thus improvement. There was thus no specific effect of TNF antagonist treatment on this ratio. This finding is in line with earlier studies, where no or small changes in lipoproteins in response to anti-TNF seemed to depend on decreased disease activity [31,44-46]. We did not study the effects of FM gain on other aspects of risk factors for CVD, such as insulin sensitivity or hypertension.

The LBM and FFM were well preserved in both treatment groups. The reason why muscle mass did not increase when disease activity decreased is probably dependent on the fact that only 20% of the patients had low muscle mass from the start in this cohort of early RA patients. The stable muscle mass indicates that the bad influence of factors known to affect muscle mass negatively - such as inflammation, disability and low levels of anabolic factors [47] - was under control in both treatment groups. These effects were probably achieved by the early intensive treatment of the disease activity and by reducing disability.

IGF-1 is important to maintain muscle mass and bone by promoting protein synthesis and inhibiting protein degradation [48,49]. The local concentration of IGF-1 is important for its anabolic actions [50], while the circulating concentration is a useful marker of nutritional status [51]. The normal serum levels of IGF-1 shown here indicate adequate energy and protein supplies.

The bone mineral was also well preserved during the two study years in the two treatment groups. The intensive treatment with powerful reduction of disease activity thus counteracted bone loss probably as a consequence of reduced inflammation. Preserved as well as increased BMD has also been reported after treatment with infliximab for 1 year in patients with established RA [52-54].

Biochemical markers of bone turnover provide a complement to measuring BMD, and although their clinical use has not yet been established they are suggested to be used in prediction of bone loss and fracture risk [55,56]. In both treatment strategies, patients had a significant decrease in markers of bone turnover. Markers of bone resorption thus decreased, with unchanged or increased ratio between P1NP and the resorption markers. These changes are consistent with those found in an uncontrolled study evaluating the effect of infliximab over 12 months in patients with established RA [52]. The reduced bone turnover suggests that both treatments have effects on inflammatory mediated bone loss.

The present study has some limitations. The study involves a rather small sample of patients but, despite this, several significant differences between the two treatment groups were found. On the contrary, because of the small sample, further differences might be undetectable. Advantages of the present study include that it is a prospective study over 21 months, which is longer than previous studies, and that we considered compliance to randomised treatment in the analyses.

## Conclusions

Treatment with infliximab increased body FM, an effect that was not achieved with the combinations of DMARDs despite similar reduction of disease activity, and thus seemed to be drug specific. Muscle mass and BMD were well preserved in both treatment groups. The increase of FM in the TNF-treated patients was not associated with an exacerbated atherogenic lipid profile. Further research is warranted, however, to determine whether the increasing FM may be associated with other risk factors for CVD, such as metabolic syndrome.

There was an increase of leptin and adiponectin concentrations in serum in both treatment groups. The increase of adiponectin suggests improved insulin sensitivity and endothelial function, which may at least partially explain the reduced frequency of CVD found when disease activity is reduced in RA, and also when anti-TNF therapy is used.

## Abbreviations

ApoA1: apolipoprotein A1; apoB: apolipoprotein B; AUC: area under the curve; BMD: bone mineral density; BMI: body mass index; 1CTP: C-terminal telopeptides of type I collagen; CTX-1: C-terminal telopeptide crosslaps; CV: coefficient of variation; CVD: cardiovascular disease; DAS28: Disease Activity Score of 28 joints; DMARD: disease-modifying antirheumatic drug; FFM: fat-free mass; FFMI: fat-free mass index; FM: fat mass; FMI: fat mass index; HAQ: Health Assessment Questionnaire; IGF-1: insulin-like growth factor 1; LBM: lean body mass; MTX: methotrexate; P1NP: procollagen type I N-terminal propeptide; RA: rheumatoid arthritis; SD: standard deviation; TNF: tumour necrosis factor.

## Competing interests

The authors declare that they have no competing interests.

## Authors' contributions

I-LE was involved in drafting the manuscript as well as the analysis and interpretation of data. BT was involved in interpretation of the data and revising the manuscript critically. KB was involved in analysing the adipokines, interpretation of the data and critically revising the manuscript. IH was involved in the study design, interpretation of data and critically revising the manuscript, as well as general supervision of the work.

## References

[B1] Rall LC Roubenoff R Rheumatoid cachexia: metabolic abnormalities, mechanisms and interventions Rheumatology (Oxford) 2004 43 1219 1223 10.1093/rheumatology/keh321 15292530

[B2] Deodhar AA Woolf AD Bone mass measurement and bone metabolism in rheumatoid arthritis: a review Br J Rheumatol 1996 35 309 322 10.1093/rheumatology/35.4.309 8624634

[B3] Giles JT Bartlett SJ Andersen RE Fontaine KR Bathon JM Association of body composition with disability in rheumatoid arthritis: impact of appendicular fat and lean tissue mass Arthritis Rheum 2008 59 1407 1415 10.1002/art.24109 18821641 PMC2670990

[B4] Abbasi F Brown BW Jr Lamendola C McLaughlin T Reaven GM Relationship between obesity, insulin resistance, and coronary heart disease risk J Am Coll Cardiol 2002 40 937 943 10.1016/S0735-1097(02)02051-X 12225719

[B5] Goodson N Marks J Lunt M Symmons D Cardiovascular admissions and mortality in an inception cohort of patients with rheumatoid arthritis with onset in the 1980 s and 1990s Ann Rheum Dis 2005 64 1595 1601 10.1136/ard.2004.034777 15843450 PMC1755282

[B6] Roubenoff R Sarcopenic obesity: the confluence of two epidemics Obes Res 2004 12 887 888 10.1038/oby.2004.107 15229325

[B7] Lipsky PE van der Heijde DM St Clair EW Furst DE Breedveld FC Kalden JR Smolen JS Weisman M Emery P Feldmann M Harriman GR Maini RN Anti-Tumor Necrosis Factor Trial in Rheumatoid Arthritis with Concomitant Therapy Study Group Infliximab and methotrexate in the treatment of rheumatoid arthritis. Anti-Tumor Necrosis Factor Trial in Rheumatoid Arthritis with Concomitant Therapy Study Group N Engl J Med 2000 343 1594 1602 10.1056/NEJM200011303432202 11096166

[B8] Marcora SM Chester KR Mittal G Lemmey AB Maddison PJ Randomized phase 2 trial of anti-tumor necrosis factor therapy for cachexia in patients with early rheumatoid arthritis Am J Clin Nutr 2006 84 1463 1472 17158431 10.1093/ajcn/84.6.1463

[B9] Metsios GS Stavropoulos-Kalinoglou A Douglas KM Koutedakis Y Nevill AM Panoulas VF Kita M Kitas GD Blockade of tumour necrosis factor-alpha in rheumatoid arthritis: effects on components of rheumatoid cachexia Rheumatology (Oxford) 2007 46 1824 1827 10.1093/rheumatology/kem291 18032540

[B10] Serelis J Kontogianni MD Katsiougiannis S Bletsa M Tektonidou MG Skopouli FN Effect of anti-TNF treatment on body composition and serum adiponectin levels of women with rheumatoid arthritis Clin Rheumatol 2008 27 795 797 10.1007/s10067-008-0855-7 18305977

[B11] Briot K Gossec L Kolta S Dougados M Roux C Prospective assessment of body weight, body composition, and bone density changes in patients with spondyloarthropathy receiving anti-tumor necrosis factor-alpha treatment J Rheumatol 2008 35 855 861 18381782

[B12] Inaba M Tanaka K Goto H Sakai S Yamada S Naka H Imanishi Y Nishizawa Y Independent association of increased trunk fat with increased arterial stiffening in postmenopausal patients with rheumatoid arthritis J Rheumatol 2007 34 290 295 17304655

[B13] van Vollenhoven RF Ernestam S Geborek P Petersson IF Coster L Waltbrand E Zickert A Theander J Thorner A Hellstrom H Teleman A Dackhammar C Akre F Forslind K Ljung L Oding R Chatzidionysiou A Wörnert M Bratt J Addition of infliximab compared with addition of sulfasalazine and hydroxychloroquine to methotrexate in patients with early rheumatoid arthritis (Swefot trial): 1-year results of a randomised trial Lancet 2009 374 459 466 10.1016/S0140-6736(09)60944-2 19665644

[B14] Arnett FC Edworthy SM Bloch DA McShane DJ Fries JF Cooper NS Healey LA Kaplan SR Liang MH Luthra HS Medsger TA Jr Mitchell DM Neustadt DH Pinals RS Schaller JG Sharp JT Wilder RL Hunder GG The American Rheumatism Association 1987 revised criteria for the classification of rheumatoid arthritis Arthritis Rheum 1988 31 315 324 10.1002/art.1780310302 3358796

[B15] Prevoo ML van't Hof MA Kuper HH van Leeuwen MA van de Putte LB van Riel PL Modified disease activity scores that include twenty-eight-joint counts. Development and validation in a prospective longitudinal study of patients with rheumatoid arthritis Arthritis Rheum 1995 38 44 48 10.1002/art.1780380107 7818570

[B16] Ekdahl C Eberhardt K Andersson SI Svensson B Assessing disability in patients with rheumatoid arthritis. Use of a Swedish version of the Stanford Health Assessment Questionnaire Scand J Rheumatol 1988 17 263 271 10.3109/03009748809098795 3187457

[B17] Physical status: the use and interpretation of anthropometry. Report of a WHO Expert Committee World Health Organ Tech Rep Ser 1995 854 1 452 8594834

[B18] Schutz Y Kyle UU Pichard C Fat-free mass index and fat mass index percentiles in Caucasians aged 18-98 y Int J Obes Relat Metab Disord 2002 26 953 960 10.1038/sj.ijo.0801856 12080449

[B19] Lunar Operator Manual, Expert-XL Software Version 1.7 1998 Madison, MA, USA: Lunar Corporation

[B20] Abernathy RP Black DR Healthy body weights: an alternative perspective Am J Clin Nutr 1996 63 3 Suppl 448S 451S 8615340 10.1093/ajcn/63.3.448

[B21] Cesari M Kritchevsky SB Baumgartner RN Atkinson HH Penninx BW Lenchik L Palla SL Ambrosius WT Tracy RP Pahor M Sarcopenia, obesity, and inflammation--results from the Trial of Angiotensin Converting Enzyme Inhibition and Novel Cardiovascular Risk Factors study Am J Clin Nutr 2005 82 428 434 16087989 10.1093/ajcn.82.2.428

[B22] Book C Karlsson MK Akesson K Jacobsson LT Early rheumatoid arthritis and body composition Rheumatology (Oxford) 2009 48 1128 1132 10.1093/rheumatology/kep165 19602478

[B23] Lewiecki EM Baim S Binkley N Bilezikian JP Kendler DL Hans DB Silverman S Report of the International Society for Clinical Densitometry 2007 Adult Position Development Conference and Official Positions South Med J 2008 101 735 739 18580720 10.1097/SMJ.0b013e31817a8b02

[B24] Ma Z Gingerich RL Santiago JV Klein S Smith CH Landt M Radioimmunoassay of leptin in human plasma Clin Chem 1996 42 942 946 8665687

[B25] Walldius G Jungner I The apoB/apoA-I ratio: a strong, new risk factor for cardiovascular disease and a target for lipid-lowering therapy - a review of the evidence J Intern Med 2006 259 493 519 10.1111/j.1365-2796.2006.01643.x 16629855

[B26] Bang P Eriksson U Sara V Wivall IL Hall K Comparison of acid ethanol extraction and acid gel filtration prior to IGF-I and IGF-II radioimmunoassays: improvement of determinations in acid ethanol extracts by the use of truncated IGF-I as radioligand Acta Endocrinol (Copenh) 1991 124 620 629 2068892 10.1530/acta.0.1240620

[B27] Hilding A Brismar K Degerblad M Thoren M Hall K Altered relation between circulating levels of insulin-like growth factor-binding protein-1 and insulin in growth hormone-deficient patients and insulin-dependent diabetic patients compared to that in healthy subjects J Clin Endocrinol Metab 1995 80 2646 2652 10.1210/jc.80.9.2646 7545695

[B28] Littell SAS System for Mixed Models 2006 2 Cary, NC, USA: SAS Institute Inc

[B29] Tilg H Moschen AR Adipocytokines: mediators linking adipose tissue, inflammation and immunity Nat Rev Immunol 2006 6 772 783 10.1038/nri1937 16998510

[B30] Fruhbeck G Gomez-Ambrosi J Muruzabal FJ Burrell MA The adipocyte: a model for integration of endocrine and metabolic signaling in energy metabolism regulation Am J Physiol Endocrinol Metab 2001 280 E827 E847 11350765 10.1152/ajpendo.2001.280.6.E827

[B31] Popa C Netea MG Radstake TR van Riel PL Barrera P van der Meer JW Markers of inflammation are negatively correlated with serum leptin in rheumatoid arthritis Ann Rheum Dis 2005 64 1195 1198 10.1136/ard.2004.032243 15731289 PMC1755600

[B32] Popa C Netea MG de Graaf J van den Hoogen FH Radstake TR Toenhake-Dijkstra H van Riel PL van der Meer JW Stalenhoef AF Barrera P Circulating leptin and adiponectin concentrations during tumor necrosis factor blockade in patients with active rheumatoid arthritis J Rheumatol 2009 36 724 730 10.3899/jrheum.080626 19273452

[B33] Straub RH Harle P Atzeni F Weidler C Cutolo M Sarzi-Puttini P Sex hormone concentrations in patients with rheumatoid arthritis are not normalized during 12 weeks of anti-tumor necrosis factor therapy J Rheumatol 2005 32 1253 1258 15996060

[B34] Kadowaki T Yamauchi T Kubota N The physiological and pathophysiological role of adiponectin and adiponectin receptors in the peripheral tissues and CNS FEBS Lett 2008 582 74 80 10.1016/j.febslet.2007.11.070 18054335

[B35] Weyer C Funahashi T Tanaka S Hotta K Matsuzawa Y Pratley RE Tataranni PA Hypoadiponectinemia in obesity and type 2 diabetes: close association with insulin resistance and hyperinsulinemia J Clin Endocrinol Metab 2001 86 1930 1935 10.1210/jc.86.5.1930 11344187

[B36] Choi HK Hernan MA Seeger JD Robins JM Wolfe F Methotrexate and mortality in patients with rheumatoid arthritis: a prospective study Lancet 2002 359 1173 1177 10.1016/S0140-6736(02)08213-2 11955534

[B37] Jacobsson LT Turesson C Gulfe A Kapetanovic MC Petersson IF Saxne T Geborek P Treatment with tumor necrosis factor blockers is associated with a lower incidence of first cardiovascular events in patients with rheumatoid arthritis J Rheumatol 2005 32 1213 1218 15996054

[B38] Komai N Morita Y Sakuta T Kuwabara A Kashihara N Anti-tumor necrosis factor therapy increases serum adiponectin levels with the improvement of endothelial dysfunction in patients with rheumatoid arthritis Mod Rheumatol 2007 17 385 390 10.1007/s10165-007-0605-8 17929130

[B39] Harle P Sarzi-Puttini P Cutolo M Straub RH No change of serum levels of leptin and adiponectin during anti-tumour necrosis factor antibody treatment with adalimumab in patients with rheumatoid arthritis Ann Rheum Dis 2006 65 970 971 10.1136/ard.2005.040857 16769786 PMC1798194

[B40] Peters MJ Watt PH Cherry L Welsh P Henninger E Dijkmans BA McInnes IB Nurmohamed MT Sattar N Lack of effect of TNFα blockade therapy on circulating adiponectin levels in patients with autoimmune disease: results from two independent prospective studies Ann Rheum Dis 2010 69 1687 1690 10.1136/ard.2009.114207 19640853

[B41] Nagashima T Okubo-Fornbacher H Aoki Y Kamata Y Kimura H Kamimura T Nara H Iwamoto M Yoshio T Okazaki H Minota S Increase in plasma levels of adiponectin after administration of anti-tumor necrosis factor agents in patients with rheumatoid arthritis J Rheumatol 2008 35 936 938 18464318

[B42] Laurberg TB Frystyk J Ellingsen T Hansen IT Jorgensen A Tarp U Hetland ML Horslev-Petersen K Hornung N Poulsen JH Flyvbjerg A Stengaard-Pedersen K Plasma adiponectin in patients with active, early, and chronic rheumatoid arthritis who are steroid- and disease-modifying antirheumatic drug-naive compared with patients with osteoarthritis and controls J Rheumatol 2009 36 1885 1891 10.3899/jrheum.080907 19684150

[B43] Frystyk J Berne C Berglund L Jensevik K Flyvbjerg A Zethelius B Serum adiponectin is a predictor of coronary heart disease: a population-based 10-year follow-up study in elderly men J Clin Endocrinol Metab 2007 92 571 576 10.1210/jc.2006-1067 17119002

[B44] Dahlqvist SR Engstrand S Berglin E Johnson O Conversion towards an atherogenic lipid profile in rheumatoid arthritis patients during long-term infliximab therapy Scand J Rheumatol 2006 35 107 111 10.1080/03009740500474578 16641043

[B45] Kiortsis DN Mavridis AK Filippatos TD Vasakos S Nikas SN Drosos AA Effects of infliximab treatment on lipoprotein profile in patients with rheumatoid arthritis and ankylosing spondylitis J Rheumatol 2006 33 921 923 16541480

[B46] Seriolo B Paolino S Sulli A Fasciolo D Cutolo M Effects of anti-TNF-alpha treatment on lipid profile in patients with active rheumatoid arthritis Ann N Y Acad Sci 2006 1069 414 419 10.1196/annals.1351.039 16855168

[B47] Engvall IL Elkan AC Tengstrand B Cederholm T Brismar K Hafstrom I Cachexia in rheumatoid arthritis is associated with inflammatory activity, physical disability, and low bioavailable insulin-like growth factor Scand J Rheumatol 2008 37 321 328 10.1080/03009740802055984 18666027

[B48] Heszele MF Price SR Insulin-like growth factor I: the yin and yang of muscle atrophy Endocrinology 2004 145 4803 4805 10.1210/en.2004-1037 15489312

[B49] McCarthy TL Centrella M Canalis E Regulatory effects of insulin-like growth factors I and II on bone collagen synthesis in rat calvarial cultures Endocrinology 1989 124 301 309 10.1210/endo-124-1-301 2909370

[B50] O'Connor JC McCusker RH Strle K Johnson RW Dantzer R Kelley KW Regulation of IGF-I function by proinflammatory cytokines: at the interface of immunology and endocrinology Cell Immunol 2008 252 91 110 10.1016/j.cellimm.2007.09.010 18325486 PMC2615236

[B51] Thissen JP Ketelslegers JM Underwood LE Nutritional regulation of the insulin-like growth factors Endocr Rev 1994 15 80 101 8156941 10.1210/edrv-15-1-80

[B52] Chopin F Garnero P le Henanff A Debiais F Daragon A Roux C Sany J Wendling D Zarnitsky C Ravaud P Thomas T Long-term effects of infliximab on bone and cartilage turnover markers in patients with rheumatoid arthritis Ann Rheum Dis 2008 67 353 357 10.1136/ard.2007.076604 17644538

[B53] Lange U Teichmann J Muller-Ladner U Strunk J Increase in bone mineral density of patients with rheumatoid arthritis treated with anti-TNF-alpha antibody: a prospective open-label pilot study Rheumatology (Oxford) 2005 44 1546 1548 10.1093/rheumatology/kei082 16263785

[B54] Vis M Havaardsholm EA Haugeberg G Uhlig T Voskuyl AE van de Stadt RJ Dijkmans BA Woolf AD Kvien TK Lems WF Evaluation of bone mineral density, bone metabolism, osteoprotegerin and receptor activator of the NFκB ligand serum levels during treatment with infliximab in patients with rheumatoid arthritis Ann Rheum Dis 2006 65 1495 1499 10.1136/ard.2005.044198 16606653 PMC1798341

[B55] Garnero P Hausherr E Chapuy MC Marcelli C Grandjean H Muller C Cormier C Breart G Meunier PJ Delmas PD Markers of bone resorption predict hip fracture in elderly women: the EPIDOS Prospective Study J Bone Miner Res 1996 11 1531 1538 10.1002/jbmr.5650111021 8889854

[B56] Garnero P Sornay-Rendu E Duboeuf F Delmas PD Markers of bone turnover predict postmenopausal forearm bone loss over 4 years: the OFELY study J Bone Miner Res 1999 14 1614 1621 10.1359/jbmr.1999.14.9.1614 10469291

